# Efficacy of a novel topical combination of esafoxolaner, eprinomectin and praziquantel against *Notoedres cati* mange in cats

**DOI:** 10.1051/parasite/2021023

**Published:** 2021-04-02

**Authors:** Martin Knaus, Balázs Capári, Mirjam Szabó, Katrin Kley, Chris Johnson

**Affiliations:** 1 Boehringer Ingelheim Vetmedica GmbH, Kathrinenhof Research Center Walchenseestr. 8–12 83101 Rohrdorf Germany; 2 Kapriol Bt. Vak Bottyán St. 1 8330 Sümeg Hungary; 3 Boehringer Ingelheim Animal Health 3239 Satellite Blvd Duluth 30096 GA USA

**Keywords:** Cat, Efficacy, Eprinomectin, Esafoxolaner, *Notoedres cati*, Mange

## Abstract

The therapeutic efficacy against notoedric mange of a topical combination of esafoxolaner, eprinomectin and praziquantel (Nexgard^®^ Combo, Boehringer Ingelheim) was evaluated in a masked, controlled clinical study including 14 cats with natural or induced *Notoedres cati* infestation. Cats were allocated randomly to two groups of seven cats each, to be administered either mineral oil (placebo control) or NexGard^®^ Combo. Each treatment was administered once as spot-on at 0.12 mL per kg body weight (representing the minimum label dosage of NexGard^®^ Combo, i.e. 1.44 mg esafoxolaner, 0.48 mg eprinomectin, and 10.0 mg praziquantel per kg body weight). Live mites were counted in skin scrapings collected within seven days prior to and 14, 27/28, 42 and 56 days after treatment to calculate the percentage efficacy of NexGard^®^ Combo based on the comparison of mean live mite counts of the two groups. Concurrently, mange lesions and clinical signs were scored to establish a clinical success valuation. No live mites were recovered from any NexGard^®^ Combo-treated cats post-treatment, indicating 100% therapeutic efficacy following a single spot-on administration of the novel antiparasitic combination*.* The clinical success valuations in the NexGard^®^ Combo-treated cats were 14.3%, 42.8%, 100% and 100% at 14, 27/28, 42 and 56 days after treatment, respectively. No health problems were observed throughout the study.

## Abstract (fr)

L’efficacité thérapeutique contre la gale notoédrique d’une association topique d’esafoxolaner, d’éprinomectine et de praziquantel (Nexgard^®^ Combo, Boehringer Ingelheim) a été évaluée dans une étude clinique contrôlée et masquée portant sur 14 chats atteints d’une infestation naturelle ou induite par *Notoedres cati*. Les chats ont été répartis au hasard en deux groupes de sept chats chacun, traités soit avec de l’huile minérale (contrôle placebo), soit avec NexGard^®^ Combo. Chaque traitement a été administré en une seule fois à raison de 0,12 mL par kg de poids corporel (représentant la posologie minimale indiquée sur l’étiquette de NexGard^®^ Combo, c’est-à-dire 1,44 mg d’esafoxolaner plus 0,48 mg d’éprinomectine plus 10,0 mg de praziquantel par kg de poids corporel). Les acariens vivants ont été comptés par grattage de peau et recueillis dans les sept jours précédant le traitement et 14, 27/28, 42 et 56 jours après le traitement pour calculer le pourcentage d’efficacité de NexGard^®^ Combo basé sur la comparaison du nombre moyen d’acariens vivants des deux groupes. Parallèlement, les lésions de la gale et les signes cliniques ont été mesurés pour établir une évaluation du succès clinique. Aucun acarien vivant n’a été retrouvé chez les chats traités par NexGard^®^ Combo après le traitement, ce qui indique une efficacité thérapeutique de 100% après une administration ponctuelle unique de la nouvelle association antiparasitaire. L’évaluation du succès clinique chez les chats traités par NexGard^®^ Combo était de 14,3 %, 42,8 %, 100 % et 100 %, respectivement 14, 27/28, 42 et 56 jours après le traitement. Aucun problème de santé n’a été observé tout au long de l’étude.

## Introduction

Feline notoedric mange is caused by infestation with burrowing mites, *Notoedres cati,* belonging to the family Sarcoptidae*.* Notoedric mange usually causes considerable discomfort and suffering in infested cats, associated with behavioural changes and severe pruritic dermatitis with crusty hyperkeratotic lesions, which may lead to self-mutilation, predisposition to secondary bacterial infection, weight loss and sometimes death. Predilected sites of infestation are the head including face, ears, neck and distal legs [2, 4, 6]. Infestation is transmitted by direct contact and may spread rapidly among cats. *Notoedres cati* occurs worldwide in domestic and wild felids, with most cases reported in Europe, India and North America. While it is primarily a parasite of felids, *N. cati* has also been reported to infest non-felid hosts, including humans [4].

Efficacious parasiticides are essential to successfully control *N. cati* infestations in domestic cats, to re-establish their health, and to protect their owners as well as wild felids in co-habitats. Macrocyclic lactone formulations [2, 12] were used in the past and were included in the first veterinary products registered and labeled for the treatment and control of feline notoedric mange (e.g., Advocate^®^/Advantage^®^ Multi, Bayer [7]; Broadline^®^, Boehringer Ingelheim [10]). The novel topical combination (NexGard^®^ Combo, Boehringer Ingelheim) contains three active ingredients, esafoxolaner, eprinomectin and praziquantel, with a broad spectrum of antiparasitic activity. Its excellent acaricidal activity is primarily attributed to the isoxazoline esafoxolaner and has been demonstrated in cats infested with ticks and ear mites [13–16].

The study reported here was conducted to evaluate the efficacy and acceptability of this novel antiparasitic product in cats in the treatment of infestations with *N. cati,* according to the current requirements for product registration.

## Materials and methods

### Compliance and ethics

The study was conducted in accordance with Guideline 7AE17a of the European Medicines Agency, Demonstration of Efficacy of Ectoparasiticides [3], and VICH Guideline 9, Good Clinical Practice [8]. Personnel involved with the evaluation of efficacy and acceptability of the product were masked in regards to the treatment assignments.

The study protocol was reviewed and approved by the Sponsor’s Institutional Animal Care and Use Committee, and the study was conducted according to Hungarian animal welfare legislation. Cats were handled with due regard for their wellbeing.

### Study animals

The study included 14 European Short-hair cats (5 males, 9 spayed females) confirmed positive for *N. cati* infestation. Infestations were either natural (4 cats) or acquired by co-housing with naturally infested cats (10 cats). The cats weighed 1.8–5.0 kg prior to treatment and were approximately 1–3 years of age. The animals were acclimated to the study facilities for at least 7 days prior to treatment administration and were housed individually during the entire study. The environmental conditions were the same for all animals which received the same commercial food, as per the manufacturer’s recommendation. Water from the public system was available *ad libitum* from bowls.

### Study design

The study used a randomized block design with blocks of two cats each based on pre-treatment mite counts. Within blocks, cats were randomly allocated to one of two groups: mineral oil (placebo control) or NexGard^®^ Combo (esafoxolaner 1.2% w/v, eprinomectin 0.4% w/v, and praziquantel 8.3% w/v). The study was done in three phases comprising four, eight and two cats, respectively, based on the availability of cats presenting mange lesions and ≥2 live mites in three skin scrapings.

### Treatment administration

Cats were treated once as spot-on at 0.12 mL per kg body weight (either placebo or NexGard^®^ Combo), representing the minimum label dosage of NexGard^®^ Combo (i.e. 1.44 mg esafoxolaner, 0.48 mg eprinomectin, and 10.0 mg praziquantel per kg body weight). The treatments were administered once (Day 0) directly on the skin, after parting the hair, in one spot in the midline of the neck between the base of the skull and the shoulder blades.

### Acceptability of treatment and general health

Animals were observed hourly for four hours following treatment and once a day throughout the study for health changes or adverse events.

### Study variables

#### Live mite counts

Skin scrapings were collected 3 or 7 days prior to Day 0, on Day 14 and every other week until Day 56 from the edges of active lesions or, if lesions regressed, from the area where active lesions were located at study commencement. Deep scrapings were made using a scalpel blade from an area of at least 1.5 cm × 1.5 cm in size. Samples were examined within eight hours of collection and live *N. cati* mites were identified based on their morphology [1] using a stereomicroscope and counted.

#### Clinical mange scores

At each skin scraping collection timepoint, the character and extent of mange lesions and the related clinical signs were evaluated and combined into individual Clinical Mange Scores (Table 1, [10]).

**Table 1:** Clinical mange scores^a^.

Score	Severity	Criteria
0	Healthy skin	Normal skin
1	Healing signs	Crusts lifted and detached easily, but hair growth not complete, slight pruritus may or may not be present
2	Mild clinical signs	Lesions locally limited, slight hair loss, moderate pruritus, thickening of skin
3	Moderate clinical signs	Lesions restricted to head region, hair loss, pruritus, thick skin, exudative (optional)
4	Severe clinical signs	Lesions covering more than head region, extensive loss of hair, severe pruritus, thick, crusty and scabby appearance of the skin, exudative (optional)

a According to Knaus et al. [10].

#### Body weight

All cats were weighed prior to treatment for dose calculation and on Day 56 to detect any body weight change over the duration of the study.

### Statistical analysis

The primary endpoint for the evaluation of therapeutic efficacy was live *N. cati* mite counts; Clinical Scores were the secondary endpoint.

The live mite counts were transformed to the natural logarithm of (count + 1) for calculation of geometric means for each treatment group. Arithmetic means for each treatment group were also calculated for each timepoint. Percent efficacy was calculated using 100 × ([*C* − *T*)/*C*], where *C* is the mean of the mineral oil (placebo control) group and *T* is the mean of the NexGard^®^ Combo group.

The log-counts of the treated group were compared to the log-counts of the untreated control group at each time point using an *F*-test adjusted for the phase and allocation blocks used to randomize the animals to the treatment groups. The MIXED procedure in SAS version 9.4 was used for the analysis, with the treatment groups listed as a fixed effect, and the phase and allocation block within phase listed as random effects.

For Clinical Scores, the proportions of each score by treatment group and by timepoint were summarized. Clinical success was determined for each post-treatment timepoint and defined as the percentage of cats per group that met these two criteria: (1) showed a lower score versus pre-treatment, and (2) were scored “0” or “1” for the respective post-treatment timepoint.

The body weight change for each treatment group over the study duration was analyzed using an *F*-test adjusted for the allocation blocks used to randomize the animals to the treatment groups. The MIXED procedure in SAS version 9.4 was used for the analysis, with the treatment groups listed as a fixed effect, and the allocation blocks listed as a random effect.

A two-sided significance level of *α* = 0.05 was used for all analyses.

## Results

No health changes other than those associated with notoedric mange were observed throughout the study.

Live *N. cati* mite counts, comparison of mite counts among the treatment groups, and percentage efficacy of the product are summarized in Table 2. For all post-treatment timepoints, live mite counts were significantly lower in the cats in the NexGard^®^ Combo group than in those in the placebo-treated control group (*p* ≤ 0.0082). No live mites were recovered from the post-treatment skin scrapings from any cats in the NexGard^®^ Combo group, demonstrating 100% therapeutic efficacy against *N. cati* mange mites of NexGard^®^ Combo administered once at the minimum label dose. According to the principles of VICH and WAAVP (World Association for the Advancement of Veterinary Parasitology), the study was considered valid because live *N. cati* mites were recovered from at least six of the seven control animals throughout the duration of the study.

**Table 2:** Live *Notoedres cati* mite counts in cats treated topically once (Day 0) either with mineral oil (placebo control) or NexGard^®^ Combo, and percentage efficacy of NexGard^®^ Combo treatment over eight weeks after treatment.

Time point	Live *Notoedres cati* mite counts				*p*-value^d^	Efficacy^e^
	Mineral oil (control)		NexGard^®^ Combo			
	NI/NG^a^	AM^b^, GM^c^, Range	NI/NG	AM, GM, Range		
Pre-treatment	7/7	31.6	7/7	24.3	0.5630	NA^f^
		22.0		17.0		
		5–91		5–67		
Day 14	7/7	10.3	0/7	0	<0.001	100%
		9.2		0		
		4–21		NA		
Day 27/28	6/7	15.3	0/7	0	0.0055	100%
		6.4		0		
		0–65		NA		
Day 42	6/7	9.4	0/7	0	0.0046	100%
		4.2		0		
		0–43		NA		
Day 56	6/7	6.9	0/7	0	0.0082	100%
		3.0		0		
		0–21		NA		

a Number of cats positive for mites/Number of cats in group.

b Arithmetic mean.

c Geometric mean (based on transformation to the natural logarithm of [count + 1]).

d *p*-value = two-sided *p*-value from the MIXED model of the NexGard^®^ Combo group and the mineral oil (control) group for mite counts.

e Efficacy = 100 × (mean mineral oil [control] − mean NexGard^®^ Combo/mean mineral oil [control]).

f Not applicable.

**Figure 1:**
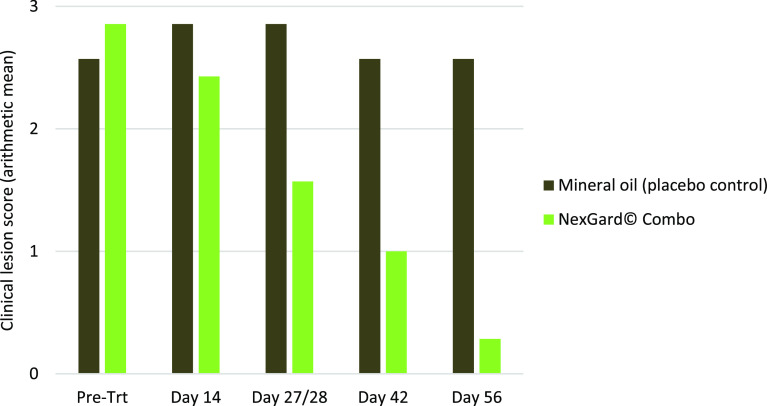
Summary of clinical mange scores per group for each observation timepoint.

Prior to treatment, all cats had Clinical Mange Scores ≥“2” (control group: 4 × “2”, 2 × “3”, 1 × “4”; NexGard^®^ Combo group: 2 × “2”, 4 × “3”, 1 × “4”). NexGard^®^ Combo treatment resulted in considerable clinical improvement from Day 27/28 onwards, while no change in the overall Clinical Mange Scores was observed in the placebo control group as shown in Figure 1. The percentage of clinical success within the NexGard^®^ Combo-treated group was 14.3%, 42.8%, 100%, and 100% for Days 14, 27/28, 42, and 56, respectively.

Over the course of the study (eight weeks), NexGard^®^ Combo-treated cats gained on average 0.13 kg or 4.7% of their pre-treatment weight, while placebo-treated control cats lost on average 0.11 kg or 3.1% of their pre-treatment weight (*p* = 0.0407).

## Discussion

The efficacy of the novel topical product NexGard^®^ Combo administered once at the minimum label dose to cats infested with *N. cati* was 100% based on mite counts. The rapid acaricidal effect of NexGard^®^ Combo treatment was also associated with a substantial improvement of clinical signs of notoedric mange, while the clinical signs remained unchanged in the control group.

The intensity of infestation of the cats included in the study in terms of pre-treatment mite counts was within the range of counts reported in previously conducted efficacy studies [7, 10], and mite counts did not differ between the placebo-treated control cats and the NexGard^®^ Combo-treated cats (*p* = 0.5630). One cat from the placebo control group became negative for mite counts from Day 28 onwards, although it was still demonstrating characteristic lesions and clinical signs of notoedric mange, with a score of “3” (Days 28 and 42) or “2” (Day 56) until the end of the study. This may indicate that *Notoedres* mites, similar to *Sarcoptes* mange mites in dogs [5], may be difficult to detect in skin scrapings because they may only be present in low numbers. Similar observations were made in a previous comparable efficacy study [10], where two of nine control cats became negative for mite counts from Day 42 onwards but demonstrated characteristic signs of mange at Days 42 and 56. The overall declining mite counts in control cats over the course of this study may have resulted from seasonal variability of mange mite infestations (the study was conducted during the months of March–July), combined with the good care of the cats, including a high quality diet and controlled laboratory conditions.

*Notoedres* mite infestation affects the health and wellbeing of cats in several ways, including extensive skin tissue damage, loss of body fluids, allergic reactions and secondary bacterial infections [11]. In the present study, mange lesions and clinical signs were completely resolved by 42 days following a single treatment with NexGard^®^ Combo. The clinical improvement was accompanied by weight gain which was significantly greater in the NexGard^®^ Combo-treated group than in the placebo control group.

Authorized products that are safe and convenient to administer and efficacious because of their sustained activity, as indicated by the plasma profile of esafoxolaner in cats administered NexGard^®^ Combo [9], are preferred by practitioners and owners. Whichever therapeutic measures are used, it is important to treat all cats that had contact with a mange affected animal to successfully break life-cycles.

## Conclusion

The results of the present study suggest that a single treatment with NexGard^®^ Combo is safe and efficacious against *Notoedres cati* mange mite infestation in cats. Clinical signs of notoedric mange resolved within 42 days following treatment.

## Competing interest

The work reported herein was funded by Boehringer Ingelheim Animal Health. The authors are current employees of Boehringer Ingelheim or external contractors. Other than that, the authors declare no conflict of interest.

This document is provided for scientific purposes only. Any reference to a brand or trademark herein is for information purposes only and is not intended for any commercial purposes or to dilute the rights of the respective owners of the brand(s) or trademark(s). Broadline^®^ and NexGard^®^ are registered trademarks of Boehringer Ingelheim Group; all other marks are the property of their respective owners.
